# Inhibition of human immunodeficiency virus type-1 (HIV-1) glycoprotein-mediated cell-cell fusion by immunor (IM28)

**DOI:** 10.1186/1743-422X-2-9

**Published:** 2005-02-11

**Authors:** Donatien Mavoungou, Virginie Poaty-Mavoungou, Marie-Yvonne Akoume, Brice Ongali, Elie Mavoungou

**Affiliations:** 1 Centre de recherche sur les pathologies hormonales, Libreville, Gabon; 2 Department of Parasitology, Institute for Tropical Medicine, University of Tübingen, Tübingen, Germany; 3 Département de Pharmacologie, Université de Montréal, Montréal, Québec, Canada; 4 Département de Physiologie, Université de Montréal, Montréal, Québec, Canada

**Keywords:** IM28, envelope glycoprotein, syncitia, fusion membrane, HIV-1

## Abstract

**Background:**

Immunor (IM28), an analog of dehydroepiandrosterone (DHEA), inhibits human immunodeficiency virus type-1 (HIV-1) by inhibiting reverse transcriptase. We assessed the ability of IM28 to inhibit the cell-cell fusion mediated by HIV envelope glycoprotein in an in vitro system. For this purpose, we co-cultured TF228.1.16, a T-cell line expressing stably HIV-1 glycoprotein envelopes, with an equal number of 293/CD4+, another T cell line expressing CD4, and with the SupT1 cell line with or without IM28.

**Results:**

In the absence of IM28, TF228.1.16 fused with 293/CD4+, inducing numerous large syncytia. Syncytia appeared more rapidly when TF228.1.16 was co-cultured with SupT1 cells than when it was co-cultured with the 293/CD4+ cell line. IM28 (1.6 – 45 μg/ml) completely inhibits cell-cell fusion. IM28 also prevented the development of new syncytia in infected cells and protected naive SupT1 cells from HIV-1 infection. Evaluation of 50% inhibitory dose (IC50) of IM28 revealed a decrease in HIV-1 replication with an IC50 of 22 mM and 50% cytotoxicity dose (CC50) as determined on MT2 cells was 75 mM giving a selectivity index of 3.4

**Conclusions:**

These findings suggest that IM28 exerts an inhibitory action on the env proteins that mediate cell-cell fusion between infected and healthy cells. They also suggest that IM28 interferes with biochemical processes to stop the progression of existing syncytia. This property may lead to the development of a new class of therapeutic drug.

## Background

The human immunodeficiency virus type-1 (HIV-1) envelope glycoprotein is composed of two subunits: a surface glycoprotein (gp120) and a trans-membrane glycoprotein (gp41). These two subunits interact with each other in a non covalent manner. Gp120 is critical for attachment to host cell CD4 receptors, whereas gp41 contains the fusion sequence. HIV and simian immunodeficiency virus (SIV) require a co-receptor in addition to CD4 for entry into cells. Primary HIV can use a broad range of co-receptor molecules, including CCR1, CCR2b, CCR3, CCR4 and CXCR4 [1-3]. However, expression of a co-receptor together with CD4 on some cell types does not confer susceptibility to infection [1]. Not all human cell types that express an appropriate co-receptor support virus replication, indicating that other factors that affect viral tropism are present. HIV-1 viral entry is inhibited in the presence of the ligands to these chemokine receptors. RANTES, MIP-1α and MIP-1β, all of which are ligands for CCR5, inhibit macrophage-tropic isolates, whereas SDF-1, the specific ligand for CXCR4, inhibits entry by T-cell-tropic isolates [4-6]. The ability of HIV-1 envelope glycoproteins to induce cell-cell fusion is an interesting property because molecules that inhibit the fusion process are possible antiviral drugs and may lead to the identification of important functional regions either on the viral glycoprotein or on cell membranes. A hydrophobic, 25-amino acid, conserved segment located at the N-terminus of gp41 and gp120/41 has been shown to be involved in the fusion reaction between the viral envelope and the host cell plasma membrane [7,8]. There is evidence suggesting that this sequence is also involved in the cytopathic process underlying HIV-1 infection of target cells [9,10]. Exposure of this hydrophobic peptide to the aqueous environment in the vicinity of the target cell initially depends on gp120/41 function [11]. This protein is activated after interacting with primary receptor CD4. This activation requires the presence of human co-factors [12,13]. According to this model, further interaction of the fusion peptide to bind membrane lipid with the cell membrane depends mainly on the ability of the peptide to bind membrane lipid components. Hence, drugs that are able to interfere with membrane proteins became relevant for the therapy of HIV, even though it is still important to inhibit virus replication. We have previously shown that IM28 can inhibit HIV-1 reverse transcriptase activity [14]. Here, we assessed its capacity to inhibit the fusion of HIV-1-infected cells to naive cells. We found that IM28 was able to inhibit cell-cell fusion in an in vitro system. We showed that IM28 significantly blocks HIV-1 glycoprotein-mediated cell-cell fusion.

## Results

**Figure 1:**
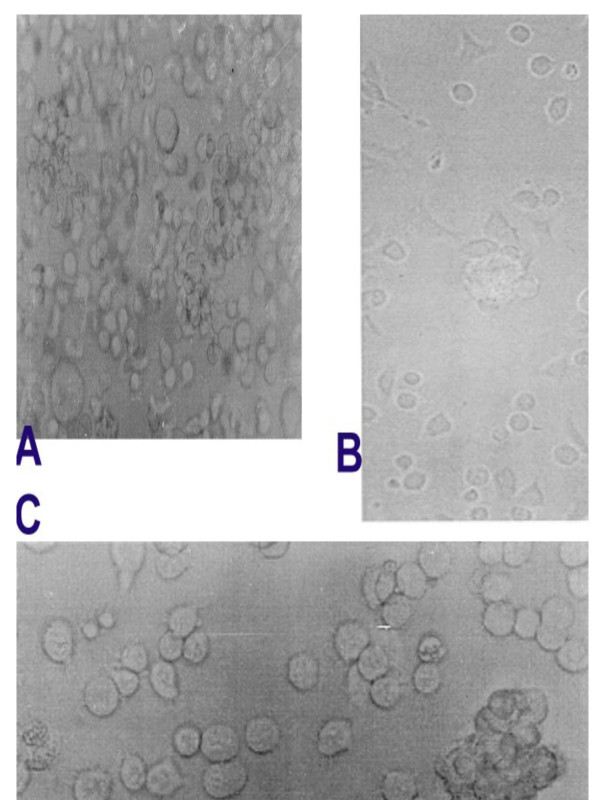
Photomicrograph of SupT1 cells co-cultivated with TF 228.1.16 cells. Cell forming syncytia are aggregated (A). In the presence of dexamethasone (B) cells are mainly exploded vs. in the presence of IM28.

We determined the concentrations of various drugs required to inhibit and to partially inhibit the fusion of TF228.1.16 and 293/CD4+ (Table 1). All these drugs decreased the percentage of surface covered by syncytia. The concentration of IM28 (6.43 μg/ml) that inhibited the formation of syncytia was similar to that of DXSF 500 000 (3.52 μg/ml) (Table 1). There were no statistical differences between the inhibitory concentrations of any of the drugs tested and IM28. To confirm these observations, we used SupT1 cells because fusion takes place more rapidly in these cells. These cells were mixed with TF22.1.16 cells in the presence or absence of dexamethasone or IM28 and fusion was examined by light microscopy after various periods of co-cultivation. In the absence of dexamethasone or IM28, TF228.1.16 cells fused with SupT1 cells, forming aggregates (Figure 1a). Infected cells were spindle-shaped with large syncytia after overnight culture (Figure 1b).

**Table 1:** Effect of drugs on fusion of TF228.1.16 cells to 293/CD4+ cells

Treatment	Effect^§^		
None	F	F	F
IM28	F (0.60)	P (1.83)	I (6.43)
Dexamethasone	F (0.48)	P (1.67)	I (5.20)
Con A	F (0.09)	P (0.22)	I (0.79)
Heparin	F (2.70)	P (7.00)	I (22.0)
Suramin	F (1.57)	P (3.90)	I (15.0)
Dextran Sulfate 10,000	F (0.02)	P (0.06)	I (0.20)
Dextran Sulfate 500,000	F (0.37)	P (1.15)	I (3.52)

^§^F = 50–60% of the surface is covered by syncytia; P = partial inhibition of fusion: < 10% of the surface is covered by syncytia; I = inhibition of syncytia formation.

TF228.1.16 cells were mixed with 293/CD4^+ ^cells (1:1 cell ratio) and transferred to a 24-well plate (10^5 ^cells per well in 200 μl of culture medium). TF228.1.16 cells and 293/CD4^+ ^cells were incubated in the presence or absence of the drug (the final concentration in μg/ml is indicated in parenthesis) for 18 h. Following co-culture, three random fields of cells were photographed (not shown) and percentage fusion was determined as previously described [10].

**Figure 2:**
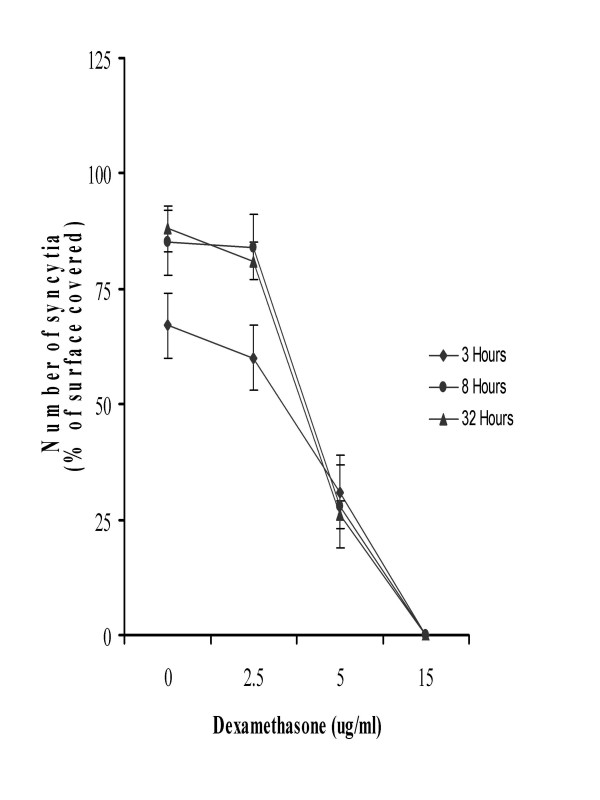
Effect of dexamethasone on fusion of TF228.1.16 cells with SupT1 cells. TF228.1.16 cells were mixed with SupT11 cells (1:1 cell ratio) and transferred to a 24-well plate (105 cells per well in 200 ml of cultured medium). After 24 h of co-culture in the presence or absence of dexamethasone (10 mg/ml), three random fields of cells were photographed and the percentage fusion was determined as described in Table 1.

**Figure 3:**
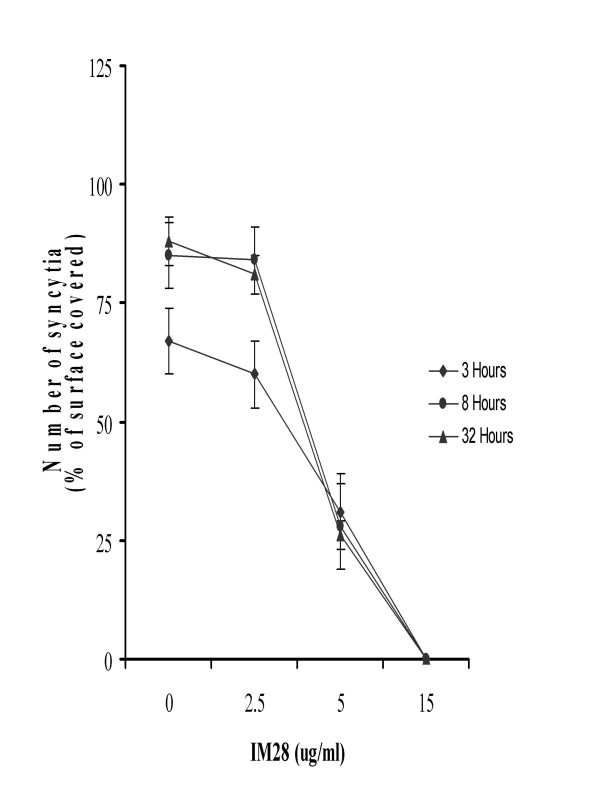
Effect of IM28 on fusion of TF228.1.16 cells with SupT1 cells. TF228.1.16 cells were mixed with SupT11 cells (1:1 cell ratio) and transferred to a 24-well plate (10^5 ^cells per well in 200 μl of cultured medium). After 24 h of co-culture in the presence or absence of corticosteroids (dexamethasone or IM28) (10 μg/ml), three random fields of cells were photographed and the percentage fusion was determined as described in Table 1.

**Figure 4:**
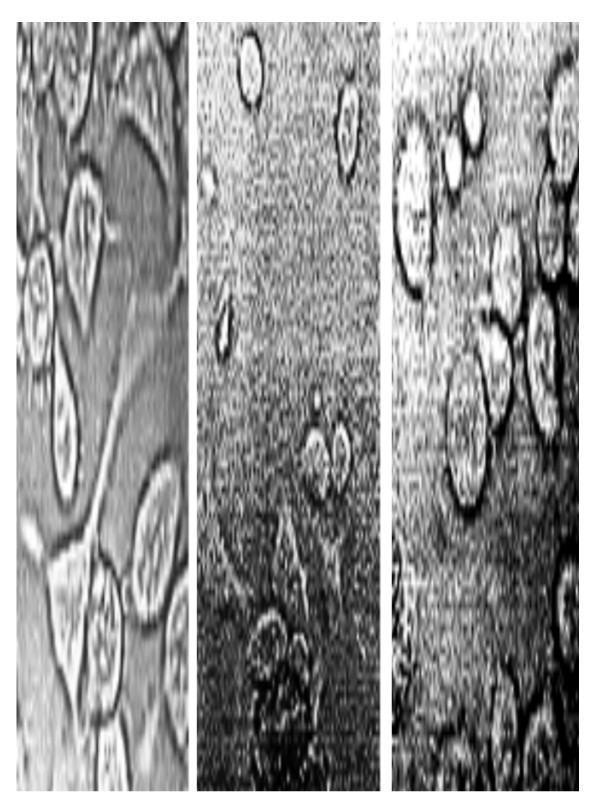
Effect of IM28 and dexamethasone on SupT1 cells co-cultured with TF228.1.16. Zoom of negative photomicrograph of SupT1 cultures co-cultivated with TF 228.1.16 cells (A) in the presence of dexamethasone (B) and IM28. Note the evident syncytia in (A) with an apparent slender shape of infected cells. Cells treated with dexamethasone were atrophic and sometimes exploded whereas cells incubated with IM28 were round.

In the presence of dexamethasone (Figure 2) or IM28 (Figure 3), the fusion of TF228.1.16 and SupT1 cells was completely inhibited in a dose-dependent manner. Indeed, in the presence of 0.5 μg/ml dexamethasone or IM28, time of incubation had no effect on syncytia formation. This concentration of dexamethasone or IM28 did not result in the lysis of existing syncytia but stopped the fusion reaction and the appearance of new syncytia (Figure 3). The time of incubation did not affect the inhibition of syncytia in the presence of dexamethasone, but did have an effect for 0.5 μg/ml IM28. In addition, the highest concentration (> 0.5 μg/ml) of both drugs completely inhibited syncytia formation. At this concentration of dexamethasone, the inhibition of syncytia was accompanied by cell death bursting (Figure 4), whereas the same concentration of IM28 did not lead to the burst (Figure 4).

To further characterize the biological effect of the drug, the 50% inhibitory dose (IC50) and the cytotoxic dose (CC50) of IM28 were evaluated and the selectivity index which is the CC50/IC50 ratio was determined. The decrease in HIV-1 replication was obtained with an IC50 of 22 mM and the CC50 as determined on MT2 cells was 75 mM giving a selectivity index of 3.4.

## Discussion

IM28 is a potent new derivative of DHEA that can stop the replication of HIV-1 by inhibiting its reverse transcriptase activity [14]. Here, we show that IM28 can also prevent and inhibit the fusion of infected cells (TF228.1.16 cells) to naïve cells including 293/CD4+ cells, which are stably transfected with human CD4 and highly susceptible to HIV-1 infection, and SupT1 cells [15,16]. The fusion of 293/CD4+ cells with TF228.1.16 cells was completely inhibited by a lower dose of IM28 than was the fusion of SupT1 cells with TF228.1.16 cells (data not shown). The fusion of TF228.1.16 cells to H4CD4+ (CD4 positive glial cell line) cells obtained by transfection of human neuroglioma cells [17] is also inhibited by IM28 (not shown). Therefore, IM28 and dexamethasone may inhibit cell-cell fusion and recombination-induced fusion mediated by the HIV env protein.

Although the precise site at which IM28 acts to inhibit cell-cell fusion remains unknown, our results suggest that IM28 fights the HIV-1 virus at a new site. It is possible that this drug interacts with phospholipase A2 (PLA2), which plays an important role in the entry of HIV virus in the host cell [18,19]. Indeed, dexamethasone, a glucocorticoid, can inhibit the HIV-1, HIV-2 and SIVmac251 envelope glycoproteins and activate PLA2. PLA2 is activated when the envelope glycoprotein interacts with CD4. Due to its local membrane-destabilizing effect, PLA2 may play an important role in preparing the cell membrane for fusion with the viral particle. Activated PLA2 hydrolyzes membrane phospholipids in the sn-2 position, producing arachidonic acid and lysophospholipids [20]. These biochemical events also have downstream effects; the membrane is destabilized locally [21,22], and arachidonic acid and lysophospholipids are generated. They are potent detergents and may favor fusion [23]. In addition, arachidonic acid is the precursor of eicosanoids, prostanoids, leukotrienes and lipoxins, which may mediate further activation [24] and PLA2-induced hydrolysis of ether lipids gives rise to paf-acether [25]. It is possible that the interaction between gp120 and CD4 specifically modifies the cell membrane locally, preparing it for fusion. We hypothesize that the gp120-CD4-co-receptor complex activates PLA2 through protein kinase C (PKC) and plays a critical role in the fusion of the membrane phospholipids of the host cells and gp41 before viral entry. Indeed, the complex formed by CD4 and p56lck acts as the major receptor for HIV-1, HIV-2 and SIV, delivering intracellular activating signals. This complex binds to the viral envelope glycoprotein gp120. Following this binding, chemokine engagement appears to be required to generate the fusion active form of the envelope protein. This may involve the formation of a gp120-CD4-chemokine receptor complex, in which engagement of the chemokine receptor is dependent on a CD4-induced conformational change in env gp120 [26-28] as previously defined for the number of parameters contributing to fusion, i.e., fusion glycoproteins and the host-cell receptors [29]. However, further investigations are required to determine the real binding site of IM28. It is possible that IM28 acts on virus replication to inhibit existing syncytia, as previously reported [14]. Therefore, although the biochemical basis of this phenomenon remains to be discovered, IM28 prevents and inhibits the cell-cell fusion induced by HIV-1, giving it additional beneficial effects. Since differential ability to incorporate or maintain envelope on the virion might account for the differences in cell-to-cell versus cell-free infections in primary isolates, further studies with a more quantitative assay available for determining fusion inhibition as previously described [33,34] may also provide us with a greater understanding of the HIV-1 envelope structure and the HIV entry process.

## Conclusion

In conclusion, our data show that IM28, a potent new analog of DHEA, is able to prevent and to inhibit cell-cell fusion, an important step at the beginning of HIV infection of naive cells, this drug seems to display the required properties for an anti-HIV drug.

## Methods

### Cell lines

Three cell lines were used: TF228.1.16, which is a BJAB cell line that stably produces functionally active HIV-1 envelope protein (BH-10 clone of HIV-1 LAI) [30]. 293/CD4+ (human embryonic kidney 293 cells which over express human CD4), obtained through the AIDS Research and Reference Reagent Program; and SupT1 cells, purchased from the American Type Culture Collection (Rockville, MD, USA).

### Reagents

DHEA, dextran-sulfate (DXSF), dexamethasone, suramin, heparin, the mannose-specific lectin concanavalin A (ConA) and Rowell Park Memorial Institute (RPMI)-1640 medium were purchased from Sigma-Aldrich (St Quentin-Fallavier, France). Cells were cultured in complete medium containing L-gltamine, penicillin, streptomycin and fetal calf serum. All these reagents were purchased from Invitrogen (Eragny, France). IM28 was produced from DHEA as specified in its data sheet (INPI 0990847; Fr2792201; Wo0106666; CRPH, Gabon).

### Fusion and syncytia assays

Cultured 293/CD4+ cells in complete medium were harvested by trypsinization. These cells (5 × 10^4^) were combined with an equal number of TF228.1.16 cells in a 24-well plate and incubated overnight at 37°C in a humidified incubator with 5% carbon dioxide as described by Moore et al. 1993 [11]. Adherent cells were fixed and stained with diff-quick (Sigma-Aldrich) and then observed under a Leitz microscope.

To examine the effect of IM28 on HIV-1 envelope glycoprotein-mediated fusion, 293/CD4+ cells were mixed with TF228.1.16 cells in the presence of IM28. As a positive control for fusion inhibition, cells were incubated in parallel with dexamethasone, ConA, heparin, suramin and dextran sulfate 10 000 or 500 000, compounds known to interfere with mannose residues of envelope glycoprotein on HIV infectivity and HIV and measles virus-induced cell fusion [31,32]. The inhibitory activity of IM28 on fusion of 293/CD4+ cells with TF228.1.16 cells is expressed as a function of concentration and was compared with the inhibitory activity of the above mentioned compounds that interact with the HIV envelope protein. Fusion was examined by light microscopy after co-cultivation for 32 h. The percentage fusion is the ratio of cell surface involved in syncytia to the total cell surface. Syncytia were defined as giant cells, with diameters more than four times bigger than those of single cells. Percentage fusion was divided into three classes: 56–00% of the surface covered by syncytial = fusion; partial inhibition of fusion: < 10% of the surface is covered by syncytial = P; inhibition of syncytia formation = I.

### Statistical analysis

Data were analyzed by one-way analysis of variance (ANOVA) followed by Dunnett' test. All analyses were performed using the Graph-Pad Prism^® ^computer program. Only *P *< 0.05 was considered significant.

## Authors' contributions

D M coordinated and participated in the design of the study, statistical analysis and the drafting of the manuscript. V P-M carried out and participated in the biological tests. M-Y A carried out and participated in the biological tests. B O carried out and participated in the biological tests. E M participated in the design of the study, carried out the biological tests and participated in the drafting of the manuscript.
